# In Response to Abiotic Stress, DNA Methylation Confers EpiGenetic Changes in Plants

**DOI:** 10.3390/plants10061096

**Published:** 2021-05-30

**Authors:** Zahida Akhter, Zhenzhen Bi, Kazim Ali, Chao Sun, Sajid Fiaz, Fasih Ullah Haider, Jiangping Bai

**Affiliations:** 1 Gansu Provincial Key Laboratory of Aridland Crop Science, Department of Crop Genetics & Breeding, College of Agronomy, Gansu Agricultural University, Lanzhou 730070, China; zahidapbmg@gmail.com (Z.A.); bizz@gasu.edu.cn (Z.B.); kazim76@gmail.com (K.A.); sunc@gsau.edu.cn (C.S.); 2 National Institute for Genomics and Advanced Biotechnology, National Agricultural Research Centre, Park Road, Islamabad 45500, Pakistan; 3 Department of Plant Breeding and Genetics, The University of Haripur, Haripur 22600, Pakistan; sfiaz@uoh.edu.pk; 4 College of Resources and Environmental Sciences, Gansu Agricultural University, Lanzhou 730070, China; fasihullahhaider281@gmail.com

**Keywords:** abiotic stresses, DNA methylation, epigenetics alterations, plant improvements

## Abstract

Epigenetics involves the heritable changes in patterns of gene expression determined by developmental and abiotic stresses, i.e., drought, cold, salinity, trace metals, and heat. Gene expression is driven by changes in DNA bases, histone proteins, the biogenesis of ncRNA, and changes in the nucleotide sequence. To cope with abiotic stresses, plants adopt certain changes driven by a sophisticated biological system. DNA methylation is a primary mechanism for epigenetic variation, which can induce phenotypic alterations in plants under stress. Some of the stress-driven changes in plants are temporary, while some modifications may be stable and inheritable to the next generations to allow them to cope with such extreme stress challenges in the future. In this review, we discuss the pivotal role of epigenetically developed phenotypic characteristics in plants as an evolutionary process participating in adaptation and tolerance responses to abiotic and biotic stresses that alter their growth and development. We emphasize the molecular process underlying changes in DNA methylation, differential variation for different species, the roles of non-coding RNAs in epigenetic modification, techniques for studying DNA methylation, and its role in crop improvement in tolerance to abiotic stress (drought, salinity, and heat). We summarize DNA methylation as a significant future research priority for tailoring crops according to various challenging environmental issues.

## 1. Introduction

The epigenetics concept was originally introduced by Waddington in the mid-20th century, by combining epigenesis and genetics for elaborating the phenotypic characteristics of plants, the result of the causal interaction between genes, and their products [1]. However, our current knowledge about molecular biology has directed us to a narrower definition that includes only the study of the molecular processes in and around DNA that control genome-related activity and phenotype, independent of the DNA sequence. It may be inherited through mitosis or meiosis and is evident from many findings that stress-induced epimutations are successfully transferred to the next generation [2,3,4,5,6]. Epigenetics has attained great success for its applications in plant breeding, where it has been used to assess the propagation of epigenetic marks across generations to improve desirable crop traits [7]. Epigenetics can be used as a potential plant breeding tool for crop improvement. Yang et al. [8] found that DNA methylation can produce epigenetic variations in response to abiotic stresses in plants. They worked on *ArabidopsisMSH1* protein. *MSH1* is one of six MutS homologs in *Saccharomyces* *cerevisiae* involved in mismatch repair but it is the only member of the family to function in the repair and maintenance of mitochondrial DNA. The RNAi suppression of *MSH1* generated phenotypic diversity for stress response gateways. After testing for seven generations, they concluded that epigenetic changes can be stable and used to speed up breeding programs enhancing plant responses to various abiotic stresses. In soybean (*Glycine max*), *MSH1* epi populations were developed by crossing with *MSH1* assimilated soybean memory lines. The resulting soybean epi lines exhibited an increase in the alteration of numerous yield-associated traits: pods per plant, seed weight, and fruit maturity time in both greenhouse and field condition experiments [9]. In *Arabidopsis* and tomato (*Solanum lycopersicum*), *MSH1* was exploited to produce rootstock epigenetic variation. The population from a grafting experiment showed improved growth vigor compared with control. A large-scale field experiment showed the consequences of *MSH1* grafting on tomato plant epigenetics over five progenies, indicating the agricultural potential of epigenetic differences and their potential to speed up crop breeding [10].

Inheritance of epigenetic markers (natural variation in DNA methylation associated with environmental changes) over generations has been reported [11,12]. Yang et al. [8] found that *MSH1*-induced epigenetic memory was stable over seven generations. The *MSH1* memory includes a different state that occurs in about 20% of plants that have experienced reprograming. Categorized by condensed growth rate, changed chlorophyll content, delay in development change and flowering, and improved stress response, *MSH1* memory is unpredictably steady, penetrant, and inherited. Genome-wide methylome analysis combined with RNAseq and network-based upgrading studies found changed circadian clock linkages, phytohormones, and stress reaction corridors that interconnect with the circadian controller. The functional HISTONE DEACETYLASE 6 and methyl-transferase MET1 are necessary for *MSH1* reprograming and therefore change the memory needs of the RNA-guided DNA methylation path. This technique of phenotypic flexibility may help plants to enhance their adaption ability during environmental alteration. Liu et al. [13] noted that heat stress memory returned back to the wild type after two generations upon withdrawal of stress. Epigenetic changes can therefore persist for many generations after plant exposure to any environmental stress. In this study, the researchers provided the idea in which genetic assistance of complex traits is divided into direct effects from core genes and unintended effects from peripheral genes acting in trans.

Epigenetic changes also have a genetic root cause, such as gene-body methylation driven by single-nucleotide polymorphisms [14]. Epigenetics plays an essential role in the understanding of natural selection, inheritance, and possibly other evolutionary processes; however, one of the difficulties in correlating phenotypic effects with specific epigenetic variations is that epigenetic and phenotypic variations may vary in natural systems [15,16,17,18]. Along with epigenetic variations, environmental factors have also been reported to promote variations; therefore, both epigenetic mechanisms and environmental factors may function as essential intercessors in mediating appropriate plant responses to adverse environmental conditions, but how much both environmental factors and epigenetic changes contribute to phenotype remains controversial [18,19,20,21]. The potential application and exploitation of epigenetics as a complementary molecular mechanism for natural selection, acclimatization, and phenotypic variations may help with plant improvement through a genetic evolutionary process that would strengthen future agriculture so that it is compatible with the environmental challenges [22,23]. Alterations in DNA play vital roles in the epigenetic regulation of expression of genes in plants. Researchers have focused on the classical epigenetic signature, 5-methylcytosine (5-mC); the field of epigenetics is receiving increased scientific attention due to the discovery of supplementary variations in DNA bases and their participation in governing gene expression. Hypothetically, each of the DNA constituents can be modified; however, only cytosine and adenine alterations are known [24]. Epigenetic modifications play an important role in the plant response mechanisms to the environment without altering DNA sequences. Cellular RNA has various chemical alterations, and these variations contribute to all features of RNA breakdown. The assembly of amendments in RNA adds a new coating to the gene guideline, leading to the development of the field of RNA epigenetics. Newly developed high-throughput sequencing tools for identifying RNA alterations have drastically advanced the practical study of RNA epitranscriptomics [25].

Although many studies have been already conducted to explore the potential effects of DNA methylation in plants, the relationship between DNA methylation and abiotic stress, i.e., heat, cold, salinity, and trace metals, has been relatively unexplored in crops. Therefore, the aim of this review is to describe and update our understanding on the epigenetic mechanisms that may help with abiotic stress adaptations. Epigenetic factors participate in abiotic stress responses, and various chromatin modifications are altered when plants are exposed to stressful environments.

## 2. DNA Methylation Associated Epigenetic Changes

Genome alteration has been proposed as a strategy to assist in the successful adaptation to severe and prolonged stresses. The epigenetic regulatory mechanism mainly comprises three levels: histone or chromatin modification, non-coding RNAs, and DNA methylation. These levels perform collectively to extend the function and regulation of genes for both normal and abnormal cellular processes [26,27].

### 2.1. DNA Methylation

DNA methylation leads to many genetically transmissible, adaptive epigenetic characteristics in plants [28]. In addition to the four nitrogenous DNA bases, small amounts of 5-methylcytosine (5-mC), N4-methylcytosine, and N6-methylcytosine are also present in DNA [5]. Among these, 5-mC is the most common and was described as the fifth DNA base, characterized and described before DNA recognition as genetic material [29]. The 5-mC nucleoside base is characterized by the covalent methylation of carbon five in the nitrogenous base of cytosine [30]. The addition of the methyl group to the DNA base provides a site for various protein complexes to bind, resulting in the modification of the histone scaffolds and the subsequent modulation of the gene expression [31]. In mammals, plants, and even prokaryotes, methylation mostly occurs in gene promoter regions, and sometimes at other transcriptional regulatory sites [32]. Recently, oxidative methylation occurring at gametogenesis, embryos, stem cells, and neurons has been studied. Many epigenetic changes have been observed during different developmental phase, which indicates the role of DNA methylation in gene expression [33]. Similarly, these mechanisms may contribute to adaptation during any environmental stress, i.e., drought, salinity, trace metals, and heat stress; however, demethylation may happen without DNA replication and in terminally differentiated cells [34,35]. Methylation sites were reported in both the heterochromatic and euchromatic regions of plant genomes [36]. Highly methylated transposable elements and other repetitive sequences are found compactly packed at heterochromatic sites, whereas euchromatic regions show comparatively less cytosine methylation [37,38]. Cytosine methylation sites can be categorized as symmetric and asymmetric sites, and methylation in plants occurs predominately at CpG sites, which are an evolutionarily conserved motif of cytosine followed by guanine attached to a phosphate [39]. Symmetric sites are found in DNA regions generally called CpG islands, which have abundant CpG and CpNpG regions, consisting of self-complementary methylable pairs of cytosines on different strands [40]. In contrast, asymmetric methylation sites consist of cytosine in any sequence and are not found in plants [41,42]. DNA demethylation of an epigenetic marker can happen with the activity of DNA glycosylase/lyase or when methylated sites are not effectively maintained [43,44,45,46,47]. In *Arabidopsis*, DNA methylation to regulate transpose on silencing, differential expression of a gene, and stable gene silencing is achieved through three different genetic pathways and deposited at CG, CHG, and CHH sequences (where H corresponds to A, T, or C) [48,49]. Methylomic and transcriptomic analyses of the genome have highlighted the variability in DNA methylation and its potential effects on the expression of genes and plant physiological characteristics [50]. Variations in plant behavior for floral morphology, fruit ripening and anthocyanin contents attributable to epialleles have been explored in many genetic studies [51]. Gene promoter methylation has been reported to control transcription and may provide a significant contribution toward necessary life phases of plants and mammals [52,53]. 5-Methylcytosine (5mC and 5fC) is an epigenetic change known to contribute to guiding gene expression in an extensive variety of biological schemes. 5mC, produced by the covalent addition of a methyl group to the fifth carbon of the pyrimidine ring of cytosine, is the most predominant epigenetic DNA alteration in the genomes of metazoans, plants, and fungi. 5mC was initially revealed in tubercle bacillus, which was shadowed by the discovery of this change in calf thymus DNA [54]. Single-molecule sequencing technology such as nanopore sequencing from Oxford Nanopore Technologies and single-molecule real-time long-read isoform sequencing from Pacific BioSciences are transforming how the transcriptome is examined. These approaches provide numerous benefits compared with the most extensively practiced high-quantity short-read RNA sequencing (RNA-Seq) methods and enable the widespread examination of transcriptomes in recognizing full-length splice isoforms and numerous other post-transcriptional measures. In addition, direct RNA-Seq offers valuable data about RNA alterations, which are lost during the PCR augmentation step in other approaches [55].

### 2.2. DNA Methyl-Transferases

DNA methyl-transferases (DNMTs) maintain pre-existing cytosine methylation patterns at previously unmethylated sites [56]. Three types of DNA methyltransferases maintain methylation post-replication: methyl-transferases 1(MET1), chromomethylase 3 (CMT3) target CG, and CHG; domains rearranged methylase (DRM2), with the help of siRNA, targets asymmetric CHH contents [57]. Interestingly, in *Arabidopsis,* DRM2 achieved de novo-methylation for all contexts of cytosine [58]. Specialized 24-nucleotide small-interfering RNA (RNA-directed DNA methylation pathway) guided DRM2 to the target loci [59]. Methyltransferase (CMT3) acts in conjunction with histone methyltransferase KYP (kryptonite/SUVH4) to maintain CHG methylation [60]. The plant ortholog of mammal DNA, (cytosine-5)-methyl-transferase 1, was shown to maintain methylation after strand replication by detection of semi-methylated CG di-nucleotide sites and denovomethylation of the corresponding non-modified CG site on the daughter strand [61]. MET1 is induced by VIM family proteins. DNMT2, homologs of which are found in mammals and plants, possesses tRNA methylase activity. The DNMT3 family comprises DNMT3a and DNMT3b genes. These methyl-transferases are expressed in undifferentiated embryonic stem cells but are down regulated following differentiation [62]. In adult somatic tissues, the expression of DNMT3 proteins is low, whereas in tumor cells, they are over-expressed and have an essential function in the hypermethylation of the CpG-rich promoter region of tumor-suppressing genes and cause inactivation of these cells [63]. At any specific site, DNA methyltransferase and demethylation enzymes control the methylation status, and it can be actively or passively demethylated [64]. Passive DNA demethylation mostly occurs due to de novo methylation inhibition or through inhibition of the maintenance of parental imprint following DNA replication [65]. Passive demethylation is induced by loss of DNA methyltransferase function [66,67]. Several currently characterized DNMT enzymes were shown to exhibit diverse but occasionally overlapping functions [68]. The strong conservation of the DNA methylation mechanism is evident through its distribution across algae, fungi, plants, invertebrates, and vertebrates [69]. This segment revealed that DNA methylation changes play a crucial role in a plant’s ability to respond to stresses. However, these changes in DNA methylation depend on the type of stress response.

## 3. Mechanisms of DNA Methylation

The symmetrical nature of CpG and CpNpG methylation facilitates their copying during DNA replication; in the case of non-symmetrical CpNpN methylation, there must be a subsequent denovomethylation for every DNA replication set [59]. It was found that most methylation occurs within CpG sites, but a notable percentage also occurs at non-CpG sites [70]. The newly synthesized, unmethylated strand creates a hemi-methylated medium with every cycle of DNA duplication that leads to the recruitment of MET1 and methylation of the opposite unmethylated CG site [71]. CHH methylation occurs through two different mechanisms. CMT2 generates H3K9me2 in the transposable elements, which are mostly present in heterochromatin [72,73]. CHH methylation relies on the actions of a self-reinforcing loop in euchromatic regions, which are the result of 24-nucleotide siRNAs directing denovomethyl-transferase for manipulating the sequences [50,64]. DNA methylation occurs with the action of various DNA methyltransferases in plants, which depends on the sequence contents; once it is established, it is further maintained byMET1 and CMT3 [65,74]. DNA demethylation can happen passively by dilution of methylation scripts by DNA duplication. The direct change of 5-methylcytosine to cytosine, as first thought, does not happen. Conversely, active DNA methylation includes oxidation of the methylated base by 10 to 11 translocations, or deamination of the methylated base. The amended nucleotide, maybe together with nearby nucleotides, is substituted by the BER corridor. New detail luminated the role of known enzymes in this procedure. They recognize base editing repair glycosylases, which may collaborate with or substitute thymine DNA glycosylase in the base removal stage and that suggest the participation of DNA destruction repair corridors other than BER in the active demethylation of DNA [75].

## 4. RNA-Mediated DNA Methylation

Plants adopt denovo-DNA methylation and gene silencing (transcriptional) using 24-nucleotide small-interfering RNAs and long non-coding RNAs in the RNA-directed DNA methylation process [67]. *Arabidopsis* mutant lines knocked out for chromatin-mediated gene-silencing small-interfering RNAs (siRNA), which provided evidence of siRNAs participation in RNA-directed DNA methylation [68]. ncRNAs formed by DNA-dependent RNA polymerases IV and V have been identified as the precursors of 24nt siRNAs necessary for RdDM; ncRNAs generated by polymerases V are directly used as scaffold RNAs [69,70,71]. The IV and V polymerases can be directed to RdDM target sites by pre-existing chromatin modifications and SHH1 protein binding to H3K9me2 by its domain, and subsequently recruits Pol IV [72]. In addition to producing 24nt siRNAs and scaffold RNAs, Pol II-mediated transcription leads to siRNA production of some RdDM loci through the involvement of the Pol IV and Pol V polymerases [72]. In contrast with Pol V, Pol II exhibits partially distinct associations with different AGO proteins [73]. Temperature plays an important role in the regulation of RNA conferred DNA methylation; it was reported that exposure to low temperature promotes VIGS, whereas high-temperature delays the process [74]. Although promoter regions undergo denovomethylation, transposable elements and some repetitive DNA elements are also silenced by this process [75]. However, the dynamic changes in epigenetic markers on stress-responsive genes make their chromatin status accessible or inaccessible and regulate the expression of stress-responsive genes at the transcriptional or post-transcriptional level.

### 4.1. MicroRNAs

Micro RNAs (miRNAs) are 20–24ntRNAs processed from longer endogenous transcripts by a dicer-like enzyme [76]. DNA-dependent RNA Pol II transcribes these either in genes targeted for post-transcriptional regulation, or in the protein-coding genes. The RNA precursors that give rise to miRNAs in plants range from 70 to more than 600 nucleotides; these are self-complementary, forming imperfect hairpin or stem-loop structures [77,78]. The function of miRNAs in DNA methylation-mediated gene regulation was also experimentally established in addition to the suppression of the expression of target genes by mRNA degradation. The involvement of miR165/166 was established in guiding the methylation of *PHB* and *PHV* transcription factor template DNA to regulate their expression during leaf surface differentiation [79].

### 4.2. Small-Interfering RNA

RNA-directed DNA methylation is a process in which siRNA-mediates denovo-DNA methylation in plants [80]. Small-interfering RNA (siRNAs), similar to miRNAs, consist of 20–24nt, and are known to play a crucial role in the heterochromatin formation processes, silencing of transposons, transgene, and mRNAs post-transcriptional regulation [81]. The siRNAs are generated through cleavage by dicer-like enzymes of long dsRNA transcribed by cis-antisense gene pairs, repetitive DNA, or non-coding transcripts [82]. These are incorporated into an AGO4 protein complex to guide the methylation machinery to the homolog DNA sequence, and thus facilitate DRM2-mediated DNA methylation. Moreover, AGO4 attaches to particular target gene promoter regions directed by enzyme-PolV-driven long non-coding RNAs to CpNpN-type asymmetric DNA methylation sites, thus suppressing their transcription [83]. The inhibition of siRNA biogenesis is a possible regulatory mechanism of the plant stress response. *Dcl2* and *Dcl3* mutants, with weakened capability for trans-activation of siRNA biogenesis, are more sensitive to genotoxic stress from exposure to methylmethane sulfonate (MMS) [84].

## 5. DNA Methylation and Tools for the Study of DNA Methylation Analysis

Several protocols are available for examining DNA methylation status. For epigenetic analysis, selection of the best method may be a difficult task when answering a specific biological query. Researchers need to explore reliable tools and techniques to address their experimental questions based on the appropriateness and feasibility of specific techniques for their sample types, the high output capabilities, and the cost-effectiveness [85,86].

### 5.1. Bisulfite-Dependent Treatment

Sodium-bisulfite functions in DNA methylation detection by converting unmethylated cytosines into uracil, while leaving 5-methyl-cytosine residues intact [87]. Sequencing by this method is useful for methylation studies. Through bisulfite treatment, DNA contributes to the formation of uracil by the deamination of cytosine and the altered residues are then read as thymine following amplification through PCR and Sanger sequencing [88]. During the last decade of the 20th century, Frommer et al. were the first to perform bisulfite conversion for the determination of 5-MEC [89]. Since these seminal studies, bisulfite conversion has become a well-established and widely used method for DNA methylation studies. However, different methods can be adopted for post-conversion PCR analysis depending on the degree of specificity and the required resolution of methylation. For the determination of methylation in the DNA molecule, cloning and sequencing are the widely used methods that may even provide single-nucleotide resolution of methylation [90]. There is one drawback to the bisulfite conversion protocol i.e., 16–20 h are required to fully replace cytosines with uracils [91]. However, high temperature can speed up the conversion of cytosine to uracil [92]. Time-course experiments demonstrated that the whole cytosine present in samples DNA can be converted to uracil by the application of high temperature (70–90 °C); even then, most of the methyl-cytosines remain unbroken [93]. Although some DNA degradation occurs in the reaction, a sufficient quantity of DNA is available for amplification and genomic sequencing. It is expected that accelerated DNA methylation analysis followed by new techniques will assist in the study of many advanced aspects of epigenetics and DNA modification for clinical applications as well as for basic research [94,95]. Another limitation that, until recently, prevented the wider application of bisulfite treatment was the requirement for a sufficient quantity of DNA for complete genome sequencing. However, a minor modification to the protocol, in which adaptor ligation is postponed until after bisulfite treatment, has allowed routine GBS with about 30ng or less of DNA; more recently, the bisulfate treatment and PCR-free methods have been devised for rapid and cost-effective methylation analysis [96,97,98]. Pyro-sequencing and MS-HRM proved to be the most suitable methods. Using pyrosequencing, every CpG in a selected area in a plant can be investigated. The instrument cost is the main drawback of this approach. MS-HRM is a simple PCR-based technique. The measurement is quick, cheap, and precise. MSRE examination is based on the precise methylation breakdown of DNA. It does not involve a bisulfite change in DNA as with the other approaches. MSRE analysis is easy to achieve, but it is not appropriate for intermediately methylated areas and is also fairly costly [99].

### 5.2. Reduced Representation Bisulfite Sequencing

Reduced representation bisulfite sequencing (RRBS) was originally developed to lower the cost associated with deciphering the mammalian methylome [100]. In the RRBS technique, Msp1 restriction enzyme digestion occurs and digested 40 to 220bps fragments are selected for bisulfite conversion and sequencing. Since most of the methylome studies focused on promoter regions, hypothetically, it would be more efficient to reduce the conversion and sequencing of non-promoter regions. RRBS was reported to provide exposure to 85% of CpG islands, which comprise 1–3% of the mammalian genome, which is only a small portion of genome sequencing [101]. RRBS is more cost-effective than WGBS and it is dependent on the successful enrichment of CpG regions, which may result in inadequate exposure of intergenic regions. However, RRBS may be the method of choice for high-throughput studies that require a cost-effective approach, and thus is widely applied in methylation patterns analysis [102]. Plant genomes profiling has also been conducted using RRBS in different crops such as *Quercus robur* [103] and *B. rapa* [104]. Platt et al. found that CpG methyl polymorphisms participate in local adaptation, either directly or through linkage to DNA regions under strong selection force [105]. Other related methods for the analysis of genomic DNA methylation are MBD-seq, Methyl Cap-seq [106,107] and using a methyl CpG-binding domain protein for DNA fragments having a high quantity of methylated CpG sites [108,109].

## 6. DNA Methylation Response to Abiotic Stress

Several studies explained DNA methylation in response to abiotic stress. Pre-exposure of plants to various abiotic stresses such as high or low temperature, high salt exposure, and deficient or flood water conditions may instigate an improved response for future stresses [110]. However, the response varies for different stresses in different plant species. Among the abiotic stresses, the major stresses are drought, heat, salt, cold, and trace metals; the majority of research focus has been on crop improvements against these stresses.

### 6.1. Adaptation to Drought

Drought stress has been an important and long-lasting research hot spot in plant biology. Under drought conditions, plants are phenotypically affected at different levels: molecular, cellular, physiological, and morphological [111]. In comparisons among *Arabidopsis* and *Zea mays* L. plants that experienced dehydration stress showed improved retention of water in the next generations or stress at later stages [109]. It has been established in many studies that repeated stress, or priming, leads plants to respond in more effectively to future challenges [112,113]. Plants exhibit dynamic methylation levels under drought stress, and epigenetic modifications are important in driving plant responses to environmental stresses [114,115,116]. Most of the epigenetic research on abiotic stress has provided shreds of evidence for stress-induced DNA methylation and demethylation either at the genome or at the specific loci level. During plant stress responses, the changes in DNA methylation patterns are sometimes associated with changes in the regulation of transcriptional genes involved in the process [117,118,119]. DNA methylation, induced by drought conditions, has been reported to contribute to adaptation to drought stress in *Oryza sativa* and in many other crops. Under drought stress conditions, a large amount of genome-site-specific variation in methylation occurs [120,121]. DNA methylation is an epigenetic mechanism in the regulation of plant gene expression that affects the plant’s developmental process, resulting in a comparatively stable plant genome during periods of external hardship [122,123]. A strong association has been found between DNA methylation and the expression of genes under drought treatment in many plants. In a DNA methylation study on *Populus trichocarpa* that under stress, the proportion of methylated cytosines was 10.04% compared with only 7.75% in the well-watered treatment [124,125]. In many other crop plants, a high proportion of drought-induced changes in DNA methylation status ore-pimutations has been reported following adaptation to long-term drought stress [83,123,126]. Numerous epigenetic processes likely function simultaneously for the effective adaptation to abiotic stress. Abscisic acid (ABA)has been established as a regulator of gene expression by inducing changes in methylation and histone acetylation [127,128]. Studies on moss *Physcomitrella patens* and Arabidopsis species reported that ABA represses gene expression through DNA methylation of the promoter regions [129,130]. In another investigation on rice cultivars exposed to drought and salt stresses, it was found that methylation extent was significantly different across the cultivars, and several methylated regions were linked with differential expression of genes necessary for abiotic stress response, with a positive correlation found among hypermethylated and small RNA [131,132]. In a study on crown gall-infected Arabidopsis, during tumor development, an epigenetic process, DNA methylation-controlled crown gall formation through ABA to mediate drought stress tolerance [133]. Global DNA methylation changes in response to recurrent drought stress were investigated in two common Greek *Medicago sativa* La. varieties (Lamia and Chaironia-Institute of Industrial and Forage Crops). The decrease in DNA methylation of stressed Lamia plants probably indicated the existence of an epigenetic mechanism that may confer drought tolerance [134,135].

### 6.2. Acclimatization to Salt

High salt accumulation in the soil is another important crop production constraint that affects 20% of the global cultivated area and strongly influences the distribution and abundance of plant species [135,136,137,138]. A genomic study on phenotypically contrasting rice lines for methylation changes under salt stress showed that hypomethylation due to salt stress is linked with the changed expression of DNA demethylases. Phenotypic variation associated with salinity tolerance may be affected due to epigenetic modulators [139,140,141]. Epigenetic changes have a composite impact on stress-inducible genes and modulate transcription factors expression [141]. A salt-tolerant *B. napus* cultivar (Exagone) and salt-sensitive *B. napus* cultivar (Toccata) were exposed to salt stress conditions; per MSAP analysis, overall DNA methylation was decreased in cv. Exagone and increased in cv. Toccata [142]. In *Arabidopsis*, a putative small RNA target area was recognized about 2.6 kb upstream of *HKT1* and was revealed to be heavily methylated [143]. The DNA methylation level in this area was reduced in the RdDM mutantrdr2, which presented the improved expression of *HKT1*, indicating that RdDM adversely controls *AtHKT1* gene expression. Parallel guiding action was also observed in wheat [144].

### 6.3. Adjustment to Heat

Heat stress is one of the predominant environmental factors posing a significant threat to food security as global warming progresses [145]. Extreme temperatures at high altitudes and in the tropical regions have been shown to influence plant growth and development including crop yield and nutritional value [146]. A study in *B. napus* revealed differences in the degree of methylation and changes in cytosine methylation patterns under heat stress in the plantlets of the two rapeseed cultivars, which were representative of heat-tolerant and heat-sensitive genotypes [147]. Both genotypes exhibited different levels of methylation under heat stress. Methylation was increased in the heat-sensitive genotype compared with the tolerant genotype; moreover, the heat-tolerant genotype showed comparatively more DNA demethylation events than the sensitive genotype. The authors found that heat exposure affected a large number of and different gene sets through changes in cytosine methylation, providing evidence that these genes mostly participate in responding to heat stress and ultimately leading to tolerance [148]. Furthermore, this study revealed that the DNA methylation alterations differed between heat-tolerant and heat-sensitive genotypes of *B. napus* in response to heat stress, which further illuminates the molecular mechanisms of the adaption to heat stress in *B. napus* [149]. The RNA-directed DNA methylation (RdDM) pathway plays an important role in the response to heat stress through the up-regulation of the epigenetic modulators DRM2, nuclear RNA polymerase NRPD1, and NRPE1 by increasing genome methylation [150]. In a transcriptome analysis investigation of the molecular pathways involved in the response to high temperature by anthers in cotton lines 84021 and H05 (*Gossypium hirsutum*), a total of 4599 differentially expressed genes were found to be involved in epigenetic modifications, carbohydrate metabolism, and regulation of plant hormone signaling [151].

### 6.4. Response to Cold Stress

Cold stress is regarded as a major environmental factor that limits agricultural expansion and crop productivity in hilly terrain [152]. Deciphering the epigenomic landscape in plants exposed to cold conditions is a rapidly developing field [153]. Methylation-sensitive amplified fragment-length polymorphism markers detected changes in cytosine methylation in the alpine subnival plant, *Chorispora bungeana,* when exposed to 4 °C chilling and −4 °C freezing stress. Rapid alterations in cytosine methylation occurred throughout the periods of chilling and freezing [154]. Comparative methylome analysis in *Populus simonii* grown under cold, osmotic, heat, and salt stresses showed condition-dependent variable cytosine methylation patterns and 1376 stress-specific differentially methylated regions (SDMRs) [155]. A new study showed that the chromatin remodeler contributes in the CBF-dependent cold tolerance in *Arabidopsis*. The pkl mutants are oversensitive to cold stress treatment [156]. In addition to histone methylation, other histone alterations play significant roles in the cold stress reaction. Histone acetylation is enhanced in the bodies of a number of cold-responsive gene [157].

### 6.5. Adaptation to Trace Metals

Heavy metal (HM) toxicity has become a major threat to sustainable crop production worldwide [158]. However, an increasing number of studies are highlighting the role of epigenetic mechanisms in the regulation of plant stress responses [159,160]. Therefore, the aim of this review was to explore and analyze the scientific literature on epigenetics as an important factor that regulates HM stress responses. In plants, specific hypomethylation of DNA was reported in clover and industrial hemp exposed to cadmium, nickel, and chromium [161]. Recent studies using the methylation-sensitive amplification polymorphism approach have demonstrated that global DNA methylation changes occur in Cd-exposed *Arabidopsis*, *O. sativa*, *Posidonia oceanic*, and *Gracilaria dura* genomes [162,163,164].

**Figure 1:**
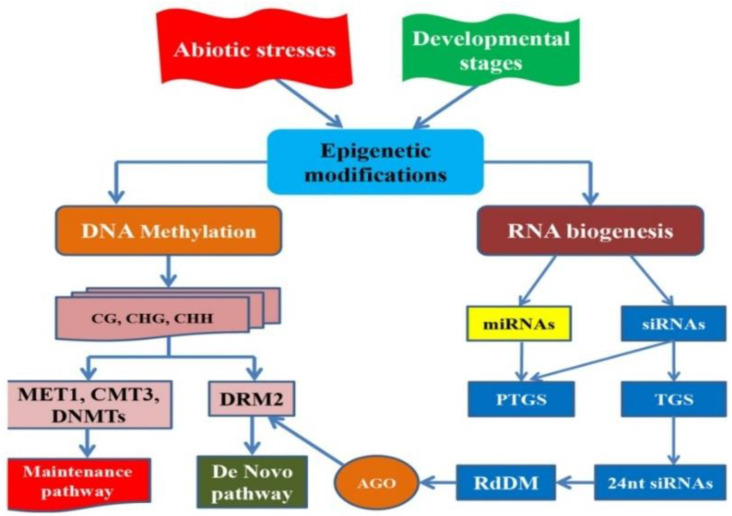
Schematic representation of regulatory network governing epigenetic modifications in response to abiotic stresses and during development in plants. The epigenetic modifications are shown here, at two levels: DNA methylation and RNA biogenesis. The boxes colors are displaying symmetry in explanations, epigenetic type, process and players involved during epigenetic modifications. The plasticity during development and abiotic stress management is displayed via routes of RNA biogenesis and DNA methylation, RNA biogenesis generating miRNAs = MicroRNAs, siRNAs = small interfering RNAs, PTGS = posttranscriptional gene silencing, TGS = transcriptional gene silencing, 24nt siRNAs = 24nucleotide small interfering RNAs, RdDM = RNA-directed DNA methylation, AGO = argonaut, CG, CHG, CHH = cytosine guanosine (where H is any base except G), MET1 = methyl transferase 1, CMT3 = chromomethylase 3, DNMTs = DNA methyltransferases, DRM2 = domains rearranged methylase.

The regulatory network involved in general in governing epigenetic modifications in response to abiotic stresses in plants is shown in Figure 1 and a brief summary of DNA methylation studies in different plant species under abiotic stresses is provided in Table 1 and Table 2.

## 7. Implementation and Prospective Applications of DNA Methylation in Plant Improvement

Recently, DNA methylation has received increased attention. DNA methylation has important functions during the response to stress in plants, differential variations of which are exhibited in different species. The pattern of DNA methylation in the genome changes during development, resulting in dynamic process involving both de novo DNA methylation and demethylation. It will be interesting to investigate the role of active DNA demethylation in other important developmental processes, such as flowering, sexual reproduction, seed germination, and programmed cell death.

### 7.1. Seed Advancement and DNA Methylation

Seed bearing in flora is coordinated by the expression of many genes, which can be synchronized by DNA-methylation [175]. It was also confirmed that DNA methylation patterns undergo active modifications during the seed development process [47]. In a study on soybean (*G. max*), 66%, 45%, and 9% of CG, CHG, and CHH sites on average, respectively, were methylated in cotyledons. CHH methylation increase was observed from 6% to 11% in cotyledons between the early and late developmental stages, respectively. A greater portion of domains rearranged methyl-transferase genes in mature seeds [175]. In maize (*Z. mays* L.), the maternally expressed gene (mee1) is established in the embryo and endosperm; upon fertilization and embryogenesis, the embryonic maternal allele of *mee1* is demethylated and remethylated, respectively, whereas the maternal *mee1* in endosperm tissue remains hypomethylated [176,177]. Another study on *B. rapa* distinctly showed that DNA methylation is mandatory for seed development [178]. Similarly, DNA hypomethylation was found in endosperm tissues comparing embryos in rice (*O. sativa*) and *A. thaliana* [179]. Different plant species exhibit variable responses for sensitivity or tolerance, to lose or interrupt DNA methylation. The RdDM pathway plays a vital role in the development of maternal somatic tissues, but not in gametophytes or zygotes [179]. The global CHH methylation level rises during seed development; subsequently, CHH methylation decreases owing to passive demethylation during germination, thereby suggesting a pivotal role of DNA methylation in seed dormancy [180,181]. It was also reported that seasonal variation in cotton (*G. hirsutum*) fiber development is linked with changes in DNA methylation [182]; Osabe et al. found that methylation changes seasonally in fibers, as well as other tissues [183]. Thus, these findings clearly indicate that DNA methylation plays a large role in gene expression and phenotypic trait development and may be used for future targeted breeding of crops.

### 7.2. Vegetative Growth and Flowering

In the life cycle of plants, flowering has key importance in vegetative and reproductive growth, being mediated by the expression of a complex gene network that is precisely controlled by flowering time. Regulation of this network is achieved by environmental signs, e.g., photoperiod, light intensity and quality, temperature, and endogenous signals instigating plant growth hormones to accomplish the task [184]. The most important factor for flowering is photoperiod, and plants are divided into three groups according to three photoperiod responsiveness: long-day, short-day, and day-neutral plants. However, some species such as *Arabidopsis* are facultative long-day (LD) plants exhibiting accelerated flowering under long-day photoperiods after vernalization, which provides an epigenetic basis for stress memory [185]. Within flowering induction signaling pathways of the leaf phloem tissue in rice, short-day (SD) photoperiods induce the expression of genes that encode globular proteins florigens to induce flowering via specific up-regulation of Hd3a and RFT1 [173]. DNA methylation has also been reported in the regulation process of flowering from floral bud development to complete flowers and seed development [186]. It was found that DNA methylation may play a vital role in the floral development process of individual male and female dioecious plants. To confirm the hypothesis of the role of DNA methylation in flowering in an experiment, DNA-methylation inhibitor 5-azac was applied on the stems of female and male basket willow trees before flower bud initiation; it accelerated the initiation of flowering and subsequent floral growth. This indicates that DNA methylation plays a significant role not only in vegetative reproductive stages but also in floral growth. In plants, the RdDM pathway primarily mediates de novo DNA methylation and siRNA screen cumbered on to argonaute-4protein. AGO4 protein interacts with domains to rearrange methyltransferase2, which catalyzes denovo DNA methylation in a sequence-free pattern [187]. In most of the flower buds, 24ntsiRNAs are predominantly expressed; approximately 0.4% may predominantly express in the buds of some vegetables, e.g., radish [176]. Mostly in non-crop plants, MSAP is used to identify variations in methylation, as in perennial ryegrass (*Lolium perenne*) [188], maize (*Z. mays* L.) [189], rapeseed (*B. napus*) [190], blackwattle (*Acacia mangium*) [180], and Japanese larch (*Larix kaempferi*) (Lamb. Carr.) [191].

### 7.3. Fruit Ripening

DNA methylation has also been reported to participate in the ripening of the fruits of many plant species [192]. In an experiment on orange (*citrus X sinensis*) fruit ripening, at the ripening stage, the orange fruit attained DNA methylation up to 30,000 genomic sites and lost ~1000 sites compared with immature orange fruits, supporting the conclusion that an increase in DNA methylation occurs during the fruit ripening process [193]. The increase in methylated sites correlates with the decreasing demethylase gene expression [194]. It was also reported that DNA hypermethylation is crucial to optimum ripening, and that hypermethylation relates to the repression of many genes, such as photosynthesis, as well as the transcriptional activation of other many genes, including loci involved in abscisic acid action [182,183,184]. Interestingly, in contrast to examples of hypermethylation during fruit ripening, active DNA demethylation was also reported as an important element participating in the control of ripening [195]. Like many other fruit crops, in the *Rosaceae* family, deferential gene expression patterns were found to play an important role in fruit setting, development, and ripening [196,197,198]. In another study, promoters of more than 200 ripening genes were identified as potential targets of SlDML2 due to their prevalent demethylation during tomato ripening [199].

## 8. Future Outlooks

DNA methylation is the main and fundamental component of epigenetic changes and an integral part of the epigenetic regulation in humans, plants, and other organisms. Although the full pathway mechanisms remain unclear, DNA methylation can modulate gene transcription, and its patterns exhibit plasticity and adaptability over time in response to environmental cues, stress, and other factors [200]. There are many developmental processes across several models and non-model organisms, a good knowledge of the epigenetic regulation would provide key insights for future research. In conclusion, epigenetic mechanisms may help plants to survive and adapt to extreme environmental conditions and selection pressures [201]. The present updated knowledge and progress in plant sciences has provided researchers with the necessary data analysis to further clarify the mechanisms of epigenetic stress responses, which may help with the development of crops with tolerance against a range of adverse abiotic factors; climate change, in particular, poses serious global challenges [202]. Epigenetic modifications are sometimes reversible but mostly provide a good source of heritable changes for quick adjustments to the environment. Some environmental stresses reportedly induce chromatin remodeling and associated changes in gene transcription. For example, vernalization states may persist in some species [203]. The benefits of the application of epigenetic engineering for agricultural production due to time requirements and use of marker-assisted selection remain unclear. So, examining epigenetic variation will help provide a comprehensive understanding of the mechanisms governing histone modification, small RNA interference, and DNA methylation, among other epigenetic modification pathways [204]. Future research in epigenetics will also help to identify the underlying mechanisms through which epigenetic markers are inherited or lost, thus providing the ability to create or remove regulatory epi-alleles that improve crop traits such as yield, sugar contents, response to different stresses, or activation or silencing of transgenes [205]. Moreover, future research will increase our ability to combine regulatory pathways, such as manipulation of transcription factor expression, with epigenetic modulation of promoter and enhancer specificity, pre-mRNA splice-site selection, and processivity by RNA polymerase, thus increasing the ability to enhance or attenuate the expression of specific genes as conditions require. It is evident from the recent progress in the field of epigenetics that future crop engineering can potentially incorporate epigenetic alterations for genetic diversity and crop improvement through novel trait selection.

## 9. Conclusions

In this review study, we briefly discussed DNA methylation, its mechanisms, and its possible role in epigenetic changes in stress tolerance development in plants. We provided some insight into DNA methylation, analysis, and its relationship with epigenetic modification and stress tolerance. Currently, epigenetics is the most promising avenue for plant scientists, and this compilation will be helpful for designing future studies to enhance genetic diversity and to manipulate regulatory pathways with epigenetics to modify or enhance the expression of specific genes for improving the stress tolerance of crops.

## Figures and Tables

**Table 1:** Recent studies on DNA methylation in diverse plant species under drought, salt, and heavy metals stress.

Stress Type	Plant	Methodology	Response	References
Drought	**Maize** *(Z. mays)*	Transcriptome, miRNA, DNA methylation analysis	Improved water retention	[115,165,166,167]
	**Mouse-ear cress** *(A. thaliana)*	Drought transcriptome analysis	Improved water retention	[168,169]
	**Rice** (*O. sativa*)	MSAP	Genome site-specific methylation deference	[170]
	**Black cottonwood** (*P. trichocarpa)*	BS-seq	Increased proportion of methylated cytosines	[120]
	**Greek** (*M. sativa*)	DNA methylation changes	Decrease in DNA methylation	[127]
	***Physcomitrella patens*** and ***Arabidopsis***	DNA methylation of gene promoters	ABA represses gene expression	[124,125]
Salt	**Rice** (*O. sativa)*	ELISA-based assay	Hypomethylation-intolerant cultivar	[130]
	**Brassica** (*B. napus*)	MSAP	Hypomethylation intolerant and hypermethylation in sensitive cultivars	[132]
	**Soybean** (*G. max*)	Expression of various transcription factors	Demethylation and hypomethylation	[136,143,159,171]
Heavy metals	**Clover**	DNA methylation analysis	Hypomethylation	[147]
	*(A. thaliana)*	MSAP	DNA methylation	[148]
	**Rice**	MSAP	DNA methylation,	[149]
	*GroceriaDura*	MSAP	DNA methylation	[150]

MSAP means “methylation-sensitive amplification polymorphism.

**Table 2:** Recent studies on DNA methylation in diverse plant species under heat and cold stress.

Stress Type	Plant	Methodology	Response	References
Cold	**Alpine**	MSAP	Cytosine methylation	[172]
	(*P. sumonii*)	Methylation	Cytosine methylation	[142]
Heat	**Rapeseed** (Brassica family)	MSAP	Increased DNA demethylation in the heat-tolerant genotype; increased DNA methylation in the heat-sensitive genotype	[137]
	**Mouse-ear cress** *(A. thaliana)*	Methylation-sensitive qPCR	Upregulation of epigenetic modulators	[138]
	**Cotton**	Regulation of anther development	DNA methylation, histone modifications	[139,173]
	**Maize** (*Z. mays*)	DNA methylation analysis	Improved heat tolerance	[174]

## Data Availability

Not applicable.

## Abbreviations

DNA: deoxyribonucleic Acid; ncRNA: non-coding RNA; tRNA: transfer RNA; CpG: Cytosine phosphodiester guanine; CMT: chromomethylase; CpNpG: cytosine phosphodiester Guanine, N is A, T, or C; DRM1: domains rearranged methyl-transferase; KYP: kryptonite; MET: DNA methyl-transferase1(MET1); VIM: variation in methylation; MTases: methyltransferases; VIGS: virus-induced gene silencing; RdDM: RNA-mediated DNA methylation; AGO: argonaute; SHH: SAWADEE homeodomain homolog 1; H3K9me2:dimethylated histone H3 lysine 9; Pol IV: RNA polymerase IV; miRNAs: microRNAs; PHB: phabulosa; PHV: phavoluta; GBS: genotyping by sequencing; RRBS: reduced representation bisulfite sequencing; Msp: methylation-specific PCR; MBD-seq: methyl-CpG-binding domain sequencing; ABA: abscisic acid; MSAP: methyl-sensitive amplification polymorphism; RdDM: RNA-directed DNA methylation; NRPD: DNA-directed RNA polymerase; BS-seq: bisulfite-seqor methyl-seq; RdDM: RNA-directed DNA methylation; LD: long-day plants; RFT1: rice flowering locus T; SD: short-day plants; siRNAs: small-interfering RNAs; SlDML: DNA demethylasegene.
